# Intermittent hand and forearm immersion in 20°C water attenuates thermal, cardiovascular, and perceptual strain in older adults during heat stress

**DOI:** 10.1113/EP092789

**Published:** 2025-07-07

**Authors:** Rachel M. Cottle, Kat G. Fisher, Olivia K. Leach, David E. Conroy, Lacy M. Alexander, W. Larry Kenney

**Affiliations:** ^1^ Department of Kinesiology The Pennsylvania State University University Park Pennsylvania USA; ^2^ Center for Healthy Aging, College of Health and Human Development The Pennsylvania State University University Park Pennsylvania USA; ^3^ School of Kinesiology University of Michigan Ann Arbor Michigan USA; ^4^ Graduate Program in Physiology The Pennsylvania State University University Park Pennsylvania USA

**Keywords:** ageing, cooling strategies, heat waves, rate–pressure product, thermal stress

## Abstract

Adults >65 years of age experience deleterious health effects during extreme heat events at a greater rate than any other age cohort. The purpose of this study was to identify the effectiveness of acute intermittent hand and forearm (H+F) water immersion and/or chronic (7 week) folic acid supplementation on reducing thermal and cardiovascular strain in older adults during heat stress. Twelve older adults (six female; 65–89 years of age) were exposed to 2 h of heat stress at 34°C and 77% relative humidity during seated rest, with and without H+F immersion, following folic acid or placebo supplementation. During immersion trials, participants immersed both H+F in cool tap water (∼20°C) for 10 min at min 60 and 90. Core temperature, skin temperature and heart rate were monitored continuously, and blood pressure and the rate–pressure product were recorded every 10 min. Data were analysed as absolute changes and as a delta from min 50 (i.e., immediately before cooling). There was no effect of folic acid on any variable (all *p* > 0.05). Absolute and delta core temperature, skin temperature and heart rate increased over time (*p* ≤ 0.041); however, H+F water immersion attenuated this increase (*p* ≤ 0.046) after the first cooling bout. Likewise, absolute and delta RPP increased over time (*p* ≤ 0.047), but absolute RPP was attenuated with H+F water immersion (*p* = 0.003). These data suggest that intermittent H+F water immersion effectively attenuates thermal and cardiovascular strain for older adults at rest during heat stress.

## Abstract (highlights)

- **What is the central question of this study?**
  We examined the effectiveness of acute intermittent hand and forearm (H+F) water immersion and chronic folic acid supplementation on reducing thermal and cardiovascular strain in older adults during heat stress.
- **What is the main finding and its importance?**
  Intermittent (H+F) water immersion in cool tap water attenuated core temperature, heart rate, rate–pressure product and thermal perception in older adults exposed to humid heat stress during seated rest. These results suggest that (H+F) water immersion is an efficacious, cost‐effective cooling strategy to reduce heat‐related morbidity and mortality for older adults.

## 1 INTRODUCTION

As global temperatures have increased over the past several decades, the frequency, duration and severity of heat waves have increased dramatically (Hansen et al., 2012; Lee, 2007). Furthermore, numerous climate models have predicted that these extreme heat events will continue to occur throughout the next coming decades (Fischer et al., 2021; Meehl & Tebaldi, 2004; Meehl et al., 2009). Epidemiological data support a strong association between extreme heat events and excess mortality, with adults >65 years of age experiencing deleterious health effects at a significantly greater rate than any other age cohort (Conti et al., 2005; Kenney et al., 2014; Semenza et al., 1999; Yip et al., 2008). Ample prior research demonstrates attenuated sweat rate and skin blood flow responses in older adults during heat stress that contributes, in part, to increased heat‐related risk in this cohort (Greaney, Alexander et al., 2015, 2015; Holowatz & Kenney, 2010; Holowatz et al., 2003, 2005; Kenney & Munce, 2003). Furthermore, researchers in our laboratory recently identified the specific combinations of temperature and humidity in which thermoregulatory health (i.e., skin blood flow and sweating response) is compromised, and therefore have significant adverse impacts for older adults (Wolf et al., 2023). These critical environments, labelled the upper limit of ‘livability’ by Vanos et al. (2023), have already been exceeded in some parts of the world and are projected to appear more frequently with global warming (Vecellio, Cottle et al., 2023). With only a 2°C increase in average temperature, ∼100 billion people globally will be exposed to prolonged (≤3 months) uncompensable humid heat stress (Vecellio, Kong et al., 2023). Therefore, given that the aged proportion of the population is growing rapidly, there will be an even greater number of people at risk during these extreme heat events (United Nations & Social Affairs, 2019).

One of the most frequently suggested safety tips during a heat wave is to stay inside and use air conditioning (American Red Cross; NationalWeatherService, 2018). Although air conditioning is an extremely effective method to reduce physiological heat strain (Bouchama et al., 2007), air conditioning use heightens global warming because it can use fossil fuels and increase greenhouse gas emissions (IEA, 2018, 2023), can increase the occurrence of power outages during extreme heat events (California Independent System Operator, 2021; Tao et al., 2022) and is not widely available for large portions of the global population during extreme heat events (IEA, 2018; Sachar et al., 2018; UNICEF, 2021). During the summer of 2021 in British Columbia, Canada, 98% of heat‐related deaths occurred indoors, and 67% of the individuals did not have access to air conditioning (BCCS, 2022). As such, low‐cost, easily accessible interventions that provide cooling are needed to reduce morbidity and mortality for older adults during extreme heat events.

Water immersion of the extremities effectively reduces and attenuates elevations in core temperature (*T*_c_) in young soldiers and firefighters following repeated exercise bouts (DeGroot et al., 2013; House, 1998; House et al., 1997; Khomenok et al., 2008; Tipton et al., 1993). A combination of both the hand and forearm (H+F) reduces thermal strain to a greater extent than hand immersion alone in water temperatures ranging from 10°C to 20°C (Giesbrecht et al., 2007). After cessation of exercise in the heat, H+F water immersion has been shown to reduce *T*_c_ by 0.21°C ± 0.2°C at a rate of 1.05°C ± 0.16°C h^−1^ in young firefighters (Selkirk et al., 2004). Elevated skin vasodilatation that occurs in response to heat stress coupled with the large surface area‐to‐mass ratio of the H+F allows for high rates of heat transfer to the cool water. As such, intermittent H+F cool‐water immersion might be a simple, effective intervention to augment heat loss acutely and reduce cardiovascular strain in older adults at rest during heat stress.

As previously mentioned, older adults have an attenuated skin blood flow response during heat stress that is largely attributable to impaired reflex vasodilatation driven by a reduction in nitric oxide bioavailability and, consequently, results in greater heat storage during heat events (Holowatz et al., 2003, 2007; Kenney, 1988; Kenny et al., 2017). Researchers in our laboratory have shown that chronic (6 week) folic acid (FA) supplementation augments skin blood flow in older adults during whole‐body heat stress by improving nitric oxide bioavailability and/or scavenging superoxide radicals, in addition to increasing the sensitivity of the microvasculature to sympathetic nervous activity (Stanhewicz et al., 2015, 2017). However, whether the augmentation in skin blood flow translates to thermoregulatory benefits in uncompensable environments where dry heat gain is minimal is likewise unexplored. Give that FA is inexpensive, easily accessible and is generally considered safe, FA supplementation might be a feasible chronic intervention to facilitate greater heat loss for older adults during extreme heat events (Campbell, 1996).

Therefore, the purpose of this study was to determine the effectiveness of acute intermittent H+F immersion in cool tap water and/or chronic FA supplementation, in addition to a synergistic effect, on reducing physiological strain in older adults at rest during uncompensable humid heat stress. We hypothesized that independently, intermittent cool‐water H+F immersion and chronic FA supplementation would attenuate the rise of *T*_c_, heart rate (HR) and rate–pressure product (RPP; i.e., an index of cardiovascular strain and myocardial oxygen uptake), resulting in an overall reduction in heat strain, attributable to enhanced convective and conductive heat transfer and increases in skin temperature (*T*_sk_) and blood flow, respectively. Furthermore, we hypothesized that the combination of intermittent H+F immersion and chronic FA supplementation would attenuate the rise of *T*_c_, HR and RPP in an additive manner.

## 2 MATERIALS AND METHODS

### 2.1 Subjects

All experimental procedures were approved in advance by the Institutional Review Board (#24458) at the Pennsylvania State University. Oral and written consents were obtained voluntarily from all subjects before participation and in accordance with the guidelines set forth by the *Declaration of Helsinki*. All testing was conducted in environmental chambers housed in Noll Laboratory at The Pennsylvania State University.

Subject characteristics are presented in Table 1. Twelve (six female; 74 ± 6 years old) subjects participated in the study. Subjects were representative of the population in this age group with respect to body size and aerobic fitness (Fryar et al., 2021; Kaminsky et al., 2015). Peak aerobic capacity (${\dot{V}}_{O_{2}\mathrm{peak}}$) was determined using open‐circuit spirometry during a graded exercise test (Bruce protocol) performed on a motor‐driven treadmill and was used as a measure of overall cardiorespiratory fitness. During the experiments, subjects wore thin, short‐sleeved cotton tee‐shirts, shorts, socks and walking/running shoes plus sports bras for the women.

**TABLE 1:** Subject characteristics.

Characteristic	Mean ± SD	Range
Age, years	74 ± 7	66–87
Height, m	1.70 ± 0.08	1.60–1.83
Weight, kg	79 ± 18	52–119
*A*_D_, m^2^	1.89 ± 0.23	1.60–2.35
*A*_D_·wt^−1^, m^2^ kg^−1^	0.025 ± 0.003	0.020–0.028
${\dot{V}}_{O_{2}\mathrm{peak}}$, mL kg^−1^ min^−1^	28.9 ± 9.0	14.6–49.6

*Note*: *n* = 12; 6 female, 6 male.

Abbreviations: *A*_D_, DuBois body surface area; A_D_·wt^−1^, body surface area‐to‐mass ratio; ${\dot{V}}_{O_{2}\mathrm{peak}}$, peak oxygen consumption.

### 2.2 Testing procedures

Participants ingested 5 mg of FA (Bio‐Tech Pharmacal) or cellulose placebo once daily for 7 weeks in a randomized, double‐blind, crossover study design. This FA treatment regimen was chosen based on previous reports suggesting that this dose and time would improve skin blood flow in older adults (Bellamy et al., 1999; Chao et al., 1999; Doshi et al., 2001; Doshi et al., 2002; Stanhewicz et al., 2015). Participants did not ingest FA or the placebo for 24 h before experimental testing to ensure that any differences were attributable to chronic and not acute effects. There was a 2 week washout period between treatments to allow for complete clearance of FA. This time frame was chosen because pharmacokinetic studies have reported the elimination half‐life of FA in the plasma to be 2.5–5 h, which would mean that ∼99.9% of the FA would be cleared after 50 h (Loew et al., 1987; Stern et al., 2011; Willems et al., 2004). The order of treatment (placebo or FA supplementation) was determined using an online random number generator. Participants were asked to stop taking a multivitamin or any other supplement that contained folate for the duration of the study. Diet was not controlled for; however, participants were encouraged to maintain the same diet across trials. Experimental visits were conducted from June to November, with the majority of participants completing all visits between July and September. All experimental visits were conducted at the same time of day for a given individual.

All participants completed four, 120 min experiments at seated rest in a controllable environmental chamber at 34°C and 77% relative humidity (rh). This environmental condition was chosen for the following reasons: (1) the majority of present and future heat waves are projected to have very high humidities (Vecellio, Cottle et al., 2023); (2) the wet‐bulb temperature associated with this environmental condition (30.4°C) has already occurred, and is projected to continue to occur, during deadly heat waves (Vecellio, Kong et al., 2023); and (3) work our laboratory has previously established the critical environmental limit at 34°C to be 67% rh for older adults at seated rest (Wolf et al., 2023), hence this environment ensured the subjects in the present study were exposed to uncompensable conditions (i.e., *T*_c_ would not equilibrate without an intervention).

Participants completed two experimental visits (one with and one without H+F water immersion) for a given treatment (i.e., a total of four experiments), separated by ≥48 h, during the 7th week of supplementation. Participants were asked to abstain from caffeine for 12 h before arrival for experiments. Upon arrival at the laboratory, participants provided a urine sample to ensure euhydration, defined as urine specific gravity ≤ 1.025 (PAL‐S; Atago, Bellevue, WA, USA). For H+F water immersion trials, participants immersed both hands and forearms into a plastic container of 20°C water for 10 min at min 60 and again at min 90 of the experiment. A water temperature of 20°C was chosen because it is approximately the temperature of cool tap water (easy access, with no electric cooling required), is tolerable by the participants and has been shown to reduce *T*_c_ following exercise in young adults (Giesbrecht et al., 2007). Two separate water containers were placed on adjustable tables in order that participants did not have to reposition themselves during water immersion. The water containers were brought into the environmental chamber immediately before the first cooling bout and remained in the chamber for the rest of the study in an effort to reflect a real‐world at‐home intervention. For control trials (no H+F immersion), participants remained at seated rest, without any additional cooling strategies, for the duration of the experiment. For a given treatment, the order of H+F immersion and non‐H+F immersion trials was determined randomly, by flipping a coin.

### 2.3 Measurements

Gastrointestinal temperature telemetry capsules (BodyCap, Hérouville‐Saint‐Clair, France) were provided for subjects to ingest 1 h before reporting to the laboratory. The *T*_c_, HR and *T*_sk_ (30 s sampling frequency; Thermochron iButton, Whitewater, WI, USA) data were continuously measured and recorded. Automated blood pressures were taken every 10 min, and RPP was calculated every 10 min.

The *T*_sk_ was measured at four sites [chest (*T*_ch_), arm (*T*_arm_), thigh (*T*_th_) and lower leg (*T*_leg_)], and a weighted mean *T*_sk_ (in degrees Celsius) was calculated (Ramanathan, 1964) as:

(1) $$T_{\mathrm{sk}}=0.3T_{\mathrm{ch}}+0.3T_{\mathrm{th}}+0.2T_{\mathrm{arm}}+0.2T_{\mathrm{leg}}.$$

RPP was calculated as:

(2) RPP=HR×SBP,

where HR is heart rate (in beats per minute) and SBP is systolic blood pressure (in millimetres of mercury). Sweat rate was determined from the loss of nude body mass on a scale accurate to ±10 g. Fluid intake was prohibited between the initial and final measurements of nude body mass. Forearm vascular conductance was measured using venous occlusion plethysmography at the beginning of and every subsequent 30 min during trials without H+F immersion (i.e., once on placebo and once following FA supplementation).

Perception of the environment [thermal, humidity (sweaty/sticky) and discomfort] and motivation for thermoregulatory behaviour were collected every 10 min using a visual analog scale and questionnaire, respectively. Participants drew a line on a 10 cm scale to represent their perception. For each scale, ‘neutral’ and ‘extremely’ were written at opposite ends of the line. For example, the thermal scale had ‘neutral’ and ‘extremely hot’ at opposite ends of the line. The distance from ‘neutral’ was recorded, and the scale was cleared after each data collection. As a proximal predictor of behaviour, at min 50 and 120, participants completed a questionnaire on their motivation for using commonly recommended interventions if they were at home, without access to air conditioning, in the same environmental conditions that they were currently experiencing (McEachan et al., 2016). This questionnaire sampled both intentions and urges to assess cognitive and affective processes that motivate health behaviour.

### 2.4 Statistical analyses

**FIGURE A1:**
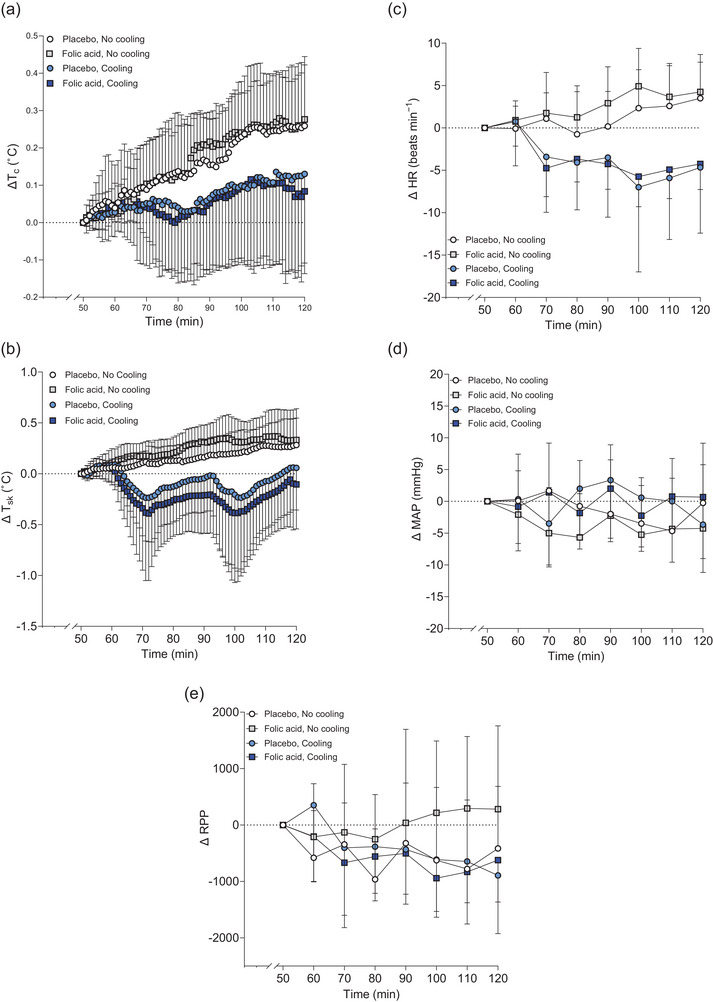
Changes from min 50 of *T*_c_ (a), *T*_sk_ (b), HR (c), MAP (d) and RPP (e) during control conditions (placebo, no cooling; open circles), after FA supplementation with no cooling (grey squares), placebo with cooling (blue circles) and after FA supplementation with cooling (blue squares) for 12 subjects with a mean age of 74 years. Data are presented as means with SD bars. FA, folic acid; Abbreviations: HR, heart rate; MAP, mean arterial pressure; RPP, rate–pressure product; *T*_c_, core temperature; *T*_sk_, skin temperature.

**FIGURE A2:**
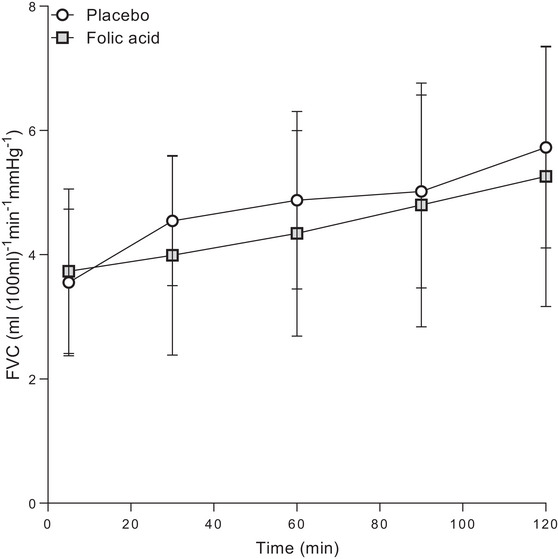
FVC during trials without (H+F) water immersion following placebo (open circles) and FA supplementation (grey squares). Data are presented as means with SD bars. Abbreviations: FA, folic acid; FVC, Forearm vascular conductance; (H+F), hand and forearm.

An a priori power analysis using an effect size of 1.3, based on previously published *T*_c_ and skin blood flow responses with these interventions (Selkirk et al., 2004; Stanhewicz et al., 2015), suggested that a minimum of eight subjects would yield sufficient statistical power (power ≥ 0.8, α = 0.05) to detect differences. Data were initially analysed to determine differences attributable to group (placebo with H+F immersion; placebo without H+F immersion; FA supplementation with H+F immersion; and FA supplementation without H+F immersion) and time on physiological and perceptual variables. Given that there was no effect of FA treatment on any physiological or perceptual variables (all *p *> 0.05; see Appendix Figures A1 and A2), data were subsequently combined (i.e., 24 observations per group) and analysed for two groups: no H+F immersion (no cooling) and H+F immersion (cooling). All ANOVAs were performed using GraphPad Prism v.9.2 (GraphPad Software, San Diego, CA, USA). A two‐way mixed‐model ANOVA was performed to examine group and time differences in *T*_c_, *T*_sk_, HR, mean arterial pressure (MAP), RPP, intent to cool off and all thermal perception scales. The *T*_c_ was analysed every 2 min, *T*_sk_ every 30 s, and HR, MAP, RPP, intent to cool off and all thermal perception scales every 10 min. Separate two‐way mixed‐model ANOVAs were performed to examine group and time differences in motivation to use each cooling strategy. A one‐way mixed‐model ANOVA was performed to examine group differences in sweat rate. Statistical significance was accepted at *p* < 0.05. When a main effect was identified, *post hoc* comparison with Tukey's corrections was performed.

## 3 RESULTS

### 3.1 Thermal and cardiovascular responses

**FIGURE 1:**
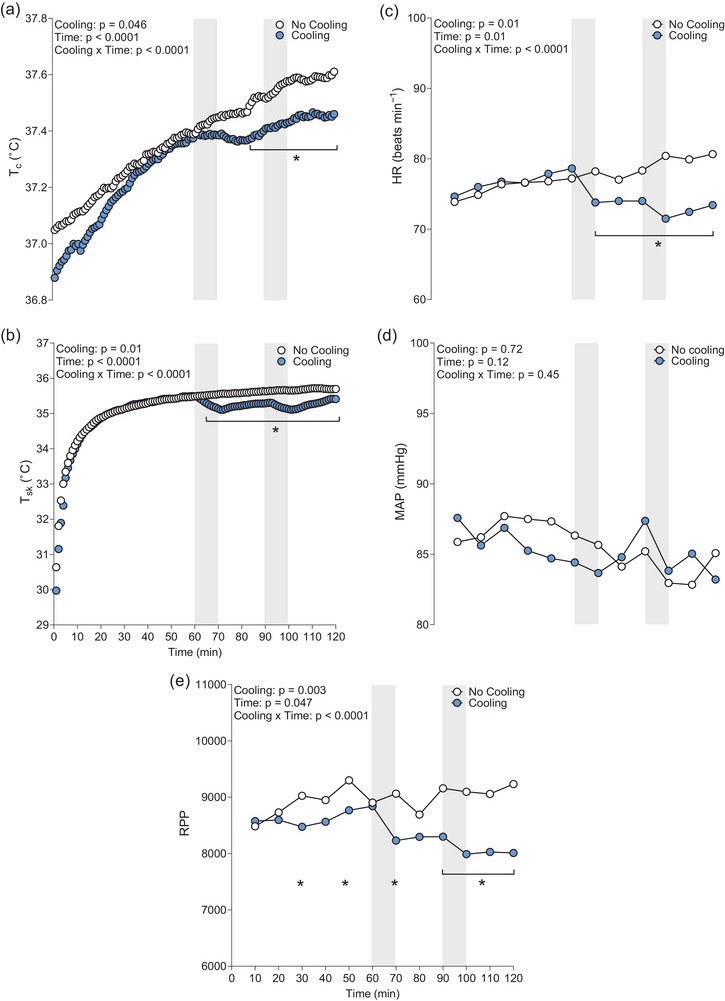
Mean responses of *T*_c_ (a), *T*_sk_ (b), HR (c), MAP (d) and RPP (e) during control trials (open circles) and cooling trials (blue circles) for 12 subjects with a mean age of 74 years. The shaded bars represent each hand + forearm immersion bout in the cooling trials. Data were analysed using a two‐way mixed‐model ANOVA. *p < 0.05 compared with control trials. Abbreviations: HR, heart rate; MAP, mean arterial pressure; RPP, rate–pressure product; *T*_c_, core temperature; *T*_sk_, skin temperature.

Absolute and delta thermal and cardiovascular responses are shown in Figures 1 (full 2 h) and 2 (beginning at min 50), respectively. The *T*_c_, *T*_sk_ and HR increased over time (main effect of time, *p* ≤ 0.041); however, H+F cooling attenuated this increase (main effect of group, *p* ≤ 0.046) when analysed from the beginning of the trial, or as a delta from min 50. There was an interaction effect (group × time) on *T*_c_ (*p* ≤ 0.001), *T*_sk_ (*p* = 0.0001) and HR (*p* = 0.0001) for both analyses. The *T*_c_, *T*_sk_ and HR were attenuated with H+F immersion, beginning at min 84, 66 and 70, respectively, when expressed as absolute data, and min 74, 66 and 70, respectively, when expressed as a delta from min 50, and continued throughout the duration of the experiment. Furthermore, absolute and delta RPP increased over time (main effect of time, *p* ≤ 0.047), but absolute RPP was attenuated with H+F cooling (main effect of group, *p* = 0.003) at min 70 and 90–120. The MAP did not change over time (main effect of time, *p* ≥ 0.12) or with the intervention (main effect of group, *p* ≥ 0.1). There was no difference in whole‐body sweat rate between cooling and no cooling trials (0.15 ± 0.14 vs. 0.17 ± 0.15 L/h; *p* = 0.33).

**FIGURE 2:**
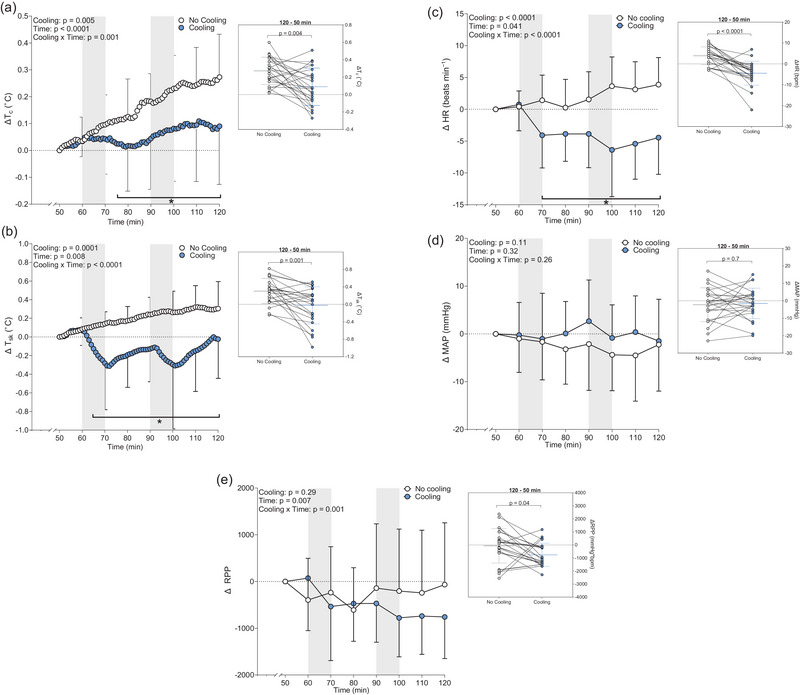
Changes from min 50 *T*_c_ (a), *T*_sk_ (b), (c), MAP (d) and RPP (e) during control trials (open circles) and cooling trials (blue circles) for 12 subjects with a mean age of 74 years. The shaded bars represent each hand + forearm immersion bout in the cooling trials. Data were analysed using a two‐way mixed‐model ANOVA. Data are presented as means with SD bars. Right‐hand panels show individual data points with connecting lines illustrating the response of an individual for the difference from min 50 to 120. *p < 0.05 compared with control trials. Abbreviations: HR, heart rate; MAP, mean arterial pressure; RPP, rate–pressure product; *T*_c_, core temperature; *T*_sk_, skin temperature.

### 3.2 Perception and motivation

**FIGURE 3:**
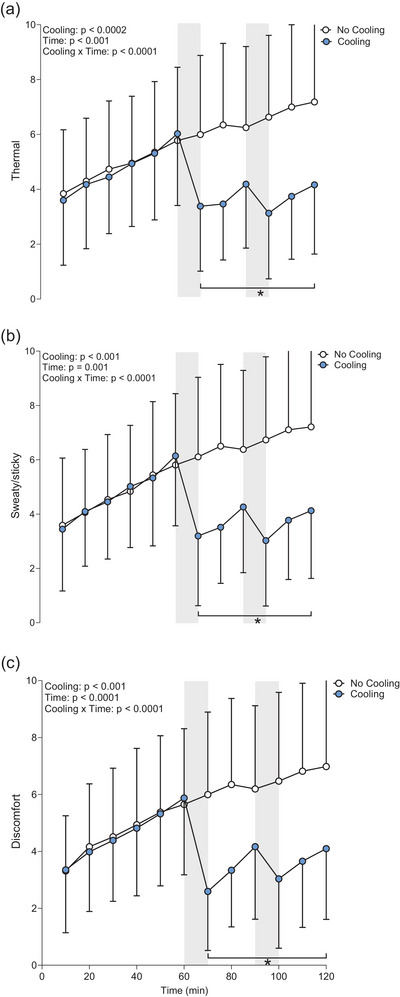
Perceptual ratings for thermal (a), sweaty/sticky sensation (b) and overall comfort (c) during control trials (open circles) and cooling trials (blue circles). The shaded bars represent each hand + forearm immersion bout in the cooling trials. Data were analysed using a two‐way mixed‐model ANOVA. Data are reported as means with SD bars. *p < 0.05 compared with control trials.

**FIGURE 4:**
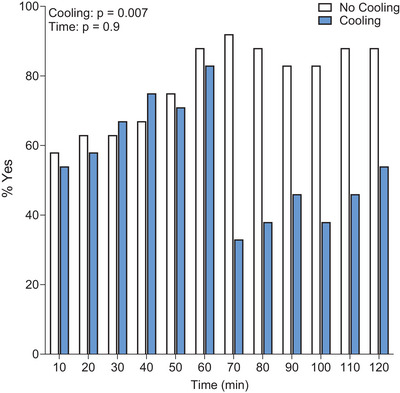
The percentage of ‘yes’ responses from participants when asked if they would try to cool themselves at each time point during control trials (open bars) and cooling trials (blue bars). Data were analysed using a two‐way ANOVA.

Environmental perception ratings are shown in Figure 3. Thermal perception, humidity (‘sweaty/sticky’) perception and perception of discomfort increased (i.e., distance from ‘neutral’) over time (main effect of time, *p* ≤ 0.001); however, H+F cooling reduced all environmental perceptions beginning with initiation (i.e., min 70) and continuing throughout the final hour (main effect of group, *p* ≤ 0.001). Furthermore, participants’ reported intent to cool themselves (Figure 4) was lower during intervention trials once H+F immersion was first initiated (*p* = 0.007).

Finally, Table 2 presents a list of various cooling strategies that participants might choose to adopt at home in hot conditions. Participants’ motivation was highest to turn on an electric fan and lowest to do nothing if they were at home in the same environmental conditions with no access to air conditioning. Furthermore, motivation to use most of the suggested cooling interventions was reduced during H+F cooling trials (*p* ≤ 0.03). There were no differences in motivation to use the suggested cooling interventions across time (*p* ≥ 0.07).

**TABLE 2:** Motivation to use recommended cooling strategies.

Strategy	50 min		120 min	
	No cooling	Cooling	No cooling	Cooling
Turn on an electric fan	9 ± 2	8 ± 2	9 ± 2	7 ± 3^*^
Drink more water	8 ± 3	7 ± 3	8 ± 3	6 ± 3^*^
Take a cold shower	5 ± 4	5 ± 4	5 ± 4	4 ± 4^*^
Place both hands and forearms in a bucket of cold water	4 ± 4	2 ± 3	4 ± 4	3 ± 3^*^
Douse the clothes you are wearing in water	2 ± 3	2 ± 3	2 ± 3	1 ± 2
Leave your home to go to an air‐conditioned environment (i.e., a mall or library)	4 ± 4	4 ± 4	5 ± 4	3 ± 4^*^
Place a wet towel on your neck	5 ± 4	5 ± 4	5 ± 4	3 ± 4
Do nothing	1 ± 1	1 ± 2	1 ± 2	1 ± 2

*Note*: Participant ratings of their motivation [ranging from 0 (not at all) to 10 (more than ever)] to use the listed cooling strategies if they were at home in the same temperature and humidity as the experimental trial with no access to air conditioning. Data were analysed using a two‐way mixed‐model ANOVA for each cooling strategy and are presented as means ± SD.

* *p* < 0.05 main effect of cooling for the listed cooling strategy.

## 4 DISCUSSION

The primary finding of this study was that acute intermittent cool‐water H+F immersion attenuated the rise in *T*_c_, HR and RPP in older adults at rest during uncompensable humid heat stress. The lower RPP was mediated by a reduction in HR, with no change in MAP. Additionally, H+F water immersion improved thermal sensation and comfort. Collectively, these findings suggest intermittent cool‐water H+F immersion is an effective, zero‐ to low‐cost intervention to reduce thermal and cardiovascular strain for older adults during humid heat stress. However, chronic folate supplementation had no independent or synergistic impact on any responses for the given environment.

It is well established that heat events are becoming longer in duration, more frequent and more severe (Hansen et al., 2012; Lee, 2007) and that older adults are the most vulnerable population during extreme heat events (Conti et al., 2005; Kenney et al., 2014; Semenza et al., 1999; Yip et al., 2008) owing, in part, to impairments in thermoregulatory function. In extreme humid environments, where the capacity for evaporation of sweat is limited, the primary cooling mechanism is through dry heat exchange and convective heat loss. Given that heat waves are becoming more humid with increasing temperatures, interventions that acutely augment convective heat loss and/or chronically improve skin blood flow responses to augment convective heat loss in older adults might reduce the detrimental effects seen in this cohort during future extreme heat.

Intermittent H+F immersion has been shown to be effective at acutely reducing thermal strain in young adults (Giesbrecht et al., 2007; House et al., 1997; Selkirk et al., 2004). For example, following exercise, *T*_c_ was reduced by 0.21°C ± 0.2°C with H+F immersion, whereas *T*_c_ increased by 0.21°C ± 0.03°C in young adults in a warm, humid (35°C, 50% rh) environment (Selkirk et al., 2004). In the present study, *T*_c_ increased by only 0.09°C ± 0.21°C from min 50 to 120 with H+F immersion, but by 0.27°C ± 0.16°C without H+F immersion. The difference in the magnitude of the effect of H+F immersion seen in previous studies with young adults compared with the present study in older adults is likely to be attributable to the difference in thermal strain between studies. In the present study, older adults were at rest, with a very low metabolic heat production (44.1 ± 8.9 W m^−2^), whereas studies with young adults had participants working at ∼30% of maximal O_2_ uptake while wearing protective clothing (i.e., firefighter or military ensembles) (Giesbrecht et al., 2007; Selkirk et al., 2004). Absolute *T*_c_ and *T*_sk_ at the start of the first cooling bout were ∼37.4°C and 36.5°C, respectively, in the present study and ∼38.3°C and 37.5°C, respectively, in previous studies (Selkirk et al., 2004), hence the gradient, and therefore magnitude, for heat loss with water immersion was lower in the present study.

However, H+F immersion in the present study still had a greater effect on *T*_c_ compared with other published reports of low‐cost individual cooling strategies implemented at rest. For example, self‐dousing and foot immersion had no effect on *T*_c_ in either a hot, humid or hot, dry environment (Morris, English et al., 2019). Furthermore, in hot, humid environments (40°C, 50% rh), fan use reduced *T*_c_ by ∼0.1°C following a 2 h exposure in young adults at rest (Morris, Gruss et al., 2019). However, in very hot environments (42°C), fan use increased convective heat gain and exacerbated thermal strain in older adults (Gagnon et al., 2016). In humid environments where ambient temperature is closer to that of *T*_sk_ (36°C), fan use has been shown to be beneficial in young adults (Ravanelli et al., 2015), although absolute changes in *T*_c_ and whether or not fan use is beneficial in these environments for older adults remains unknown. Regardless, given that heat waves are typically accompanied with power outages, our data suggest that intermittent H+F cool‐water immersion might be a more accessible, effective cooling modality to attenuate thermal strain for older adults.

The reported effects of H+F immersion on cardiovascular responses have been equivocal, with some studies reporting no benefit (Giesbrecht et al., 2007) and others reporting ≤33% reduction in HR (Khomenok et al., 2008; Selkirk et al., 2004). Here, we show that H+F immersion reduced resting HR after the first cooling bout, and HR remained lower for the duration of the study in older adults. At the end of the trial, mean HR was 8 beats min^−1^ lower with H+F immersion compared with control conditions. H+F immersion has been shown to reduce sweat rate by 22% (Giesbrecht et al., 2007) or 0.45 L h^−1^ (Selkirk et al., 2004); however, there was no difference in sweat rate with H+F immersion in the present study. This was probably attributable to the very low metabolic rate (rest) and moderate *T*_c_ and *T*_sk_ resulting in a small stimulus for the sweating response. Although, given that *T*_c_ was reduced with H+F immersion, it is likely that the skin blood flow requirement was attenuated, which resulted in a reduction in HR. Furthermore, the attenuated skin blood flow requirement might have resulted in less peripheral pooling in the compliant cutaneous circulation during seated rest, which would help to maintain stroke volume and allow for HR to be reduced. Given that MAP did not change throughout the trials, this reduction in HR primarily drove the reduction in RPP. Given that RPP reflects myocardial oxygen consumption and is used as an index of cardiovascular strain (Klabunde, 2011), our findings suggest that H+F immersion might reduce cardiovascular strain in older adults.

A potential chronic preventive measure to mitigate thermal strain in older adults is to augment the skin blood flow response to heat stress. Work in our laboratory has shown that chronic (6 week) FA supplementation increased cutaneous vasodilatation during whole‐body heat stress (Stanhewicz et al., 2015, 2017). However, FA supplementation has been shown to have no effect on *T*_sk_ in extreme heat (Gagnon et al., 2018), although in that study ambient temperature was held constant at 42°C, providing a large skin‐to‐air gradient that favoured dry heat gain, potentially minimizing any effect that would have been seen with FA supplementation on skin blood flow or *T*_sk_. Therefore, we hypothesized that in humid environments where ambient temperature was closer to *T*_sk_ (i.e., 34°C), the augmented skin blood flow response with FA supplementation might slightly increase *T*_sk_ above ambient temperature to allow for convective heat loss. However, forearm blood flow and *T*_sk_ were not different with FA supplementation in the present study. Although speculative, the lack of increase in forearm blood flow, and therefore *T*_sk_, might have been a result of the relatively mild stimulus to drive an increase in whole‐limb blood flow, because the change in *T*_c_ was moderate (∼0.6°C) throughout each trial. In support of this idea, forearm blood flow was increased during whole‐body heat stress with water‐perfused suits in older adults following supplementation with sapropterin (Stanhewicz et al., 2013). However, this augmented forearm blood flow response did not occur until oral temperature increased by ∼0.6°C although cutaneous blood flow was greater at baseline and throughout a 1°C increase with sapropterin (Stanhewicz et al., 2013). Therefore, it might be possible that the effect and/or benefit of FA supplementation is not evident until a greater degree of thermal strain occurs than seen in the present study.

In addition to physiological responses, regulation of body temperature also occurs through behavioural responses driven by thermal (dis)comfort and sensation (Schlader et al., 2011a). Behavioural thermoregulation is arguably the most effective response, especially in environments that are uncompensable, where autonomic thermoeffector responses alone cannot maintain thermal balance (Hardy, 1971). Behavioural responses in the heat are related to physiological input, because behaviour is driven by changes in both *T*_sk_ (predominantly in the periphery and face) and *T*_c_ (Schlader et al., 2011a, 2011b). Therefore, unsurprisingly, as *T*_sk_ was lowered with H+F immersion in the present study, participants’ thermal comfort and sensation improved, and their intention to cool themselves further was lower. As *T*_sk_ began to rise after the first H+F immersion, the reported intention to cool subsequently increased, but was again lowered when *T*_sk_ was reduced following the second H+F immersion bout. Interestingly, the percentage of participants who reported that they desired to cool themselves plateaued at the same time *T*_sk_ began to plateau, although *T*_c_ continued to rise. These findings support the idea that *T*_sk_ is the strongest predictor for behavioural input and suggest that H+F immersion effectively improves thermal comfort/sensation.

To gain a better understanding of which cooling strategies older adults might prefer to use during extreme heat events, we recorded both cognitive and affective processes that motivate health behaviour. Participants reported their intention and urge to implement a variety of suggested cooling strategies if they were at home in the same hot environmental conditions as the experimental trial. Given that (1) the regions that will experience the most extreme heat events have less access to air conditioning (IEA, 2019; Rastogi et al., 2023), (2) power outages commonly occur during heat waves (California Independent System Operator, 2021; Tao et al., 2022), and (3) the majority of older adults who die during heat waves do not have air conditioning (Fouillet et al., 2006; Service, 2022), we asked participants to respond as if they did not have air conditioning at home. Participants’ motivation was highest for turning on an electric fan, followed by drinking more water.

According to modelling data, fan use might be beneficial in lowering *T*_c_ for older adults in the environment used here (Jay et al., 2015); however, relying on fan use might be problematic in the event of a power outage or in regions that do not have electricity. Furthermore, although drinking water might temporarily improve thermal sensation/comfort and maintain hydration status to attenuate cardiovascular strain, it would probably not be effective in maintaining a reduction in *T*_c_ during uncompensable heat stress, because heat gain would still exceed heat loss. Participants had little motivation to use other cooling strategies recommended by the National Weather Service, such as spending time in a public air‐conditioned environment, such as a mall or library, taking a cool shower or self‐dousing. Although participants did not report high levels of motivation to use H+F immersion, motivation to use the majority of cooling strategies was lower in H+F compared with non‐H+F immersion trials. Although speculative, this might be because participants felt cooler at the end of trials with H+F immersion, leaving them with less motivation to cool because they were satisfied with their thermal comfort.

## 5 CONCLUSION

In summary, the present investigation aimed to determine the effectiveness of both an acute and a chronic feasible, cost‐effective and sustainable cooling strategy to reduce thermal and cardiovascular strain in older adults in an uncompensable hot, humid environment. There were no independent or synergist effects of chronic FA supplementation on thermal or cardiovascular responses. However, we demonstrated that acute intermittent H+F immersion in cool tap water provided a periodic reduction in *T*_sk_ and a sustained reduction in *T*_c_ during uncompensable heat stress. This reduction in *T*_c_ might be sufficient to prolong the time to reach a *T*_c_ associated with heat illnesses in older adults. Furthermore, our data suggest that this simple cooling strategy effectively reduced cardiovascular strain in older adults during heat stress, as evidenced by a lower and sustained HR and RPP response with H+F immersion. In addition, thermal sensation and comfort were greatly improved with cold‐water H+F immersion. This resulted in participants feeling more satisfied with their thermal comfort, because fewer reported the need to implement additional cooling after H+F immersion. Lastly, we identified widely recommended cooling strategies that older adults would be likely to adopt at home during a heat wave. The findings from this study provide useful information that can be used for evidence‐based safety interventions and heat action plans to mitigate the impact of severe heat events on health outcomes in older adults.

## AUTHOR CONTRIBUTIONS

Rachel M. Cottle, David E. Conroy, Lacy M. Alexander and W. Larry Kenney conceived and designed research; Rachel M. Cottle, Kat G. Fisher and Olivia K. Leach performed experiments; Rachel M. Cottle analysed data; Rachel M. Cottle, Kat G. Fisher, Olivia K. Leach, David E. Conroy, Lacy M. Alexander and W. Larry Kenney interpreted results of experiments; Rachel M. Cottle prepared figures; Rachel M. Cottle drafted the manuscript; Rachel M. Cottle, Kat G. Fisher, Olivia K. Leach, David E. Conroy, Lacy M. Alexander and W. Larry Kenney edited and revised the manuscript; Rachel M. Cottle, Kat G. Fisher, Olivia K. Leach, David E. Conroy, Lacy M. Alexander and W. Larry Kenney approved the final version of manuscript and agree to be accountable for all aspects of the work in ensuring that questions related to the accuracy or integrity of any part of the work are appropriately investigated and resolved. All persons designated as authors qualify for authorship, and all those who qualify for authorship are listed.

## CONFLICT OF INTEREST

None declared.

## Data Availability

Data will be made available upon reasonable request.
